# Parental Knowledge of Children's Developmental Milestones in Riyadh, Saudi Arabia

**DOI:** 10.1155/2020/8889912

**Published:** 2020-10-22

**Authors:** Abdulrahman S. Aldayel, Abdulaziz A. Aldayel, Ahmed M. Almutairi, Hamad A. Alhussain, Sultan A. Alwehaibi, Talal A. Almutairi

**Affiliations:** ^1^College of Medicine, Imam Mohammad Ibn Saud Islamic University (IMSIU), Riyadh, Saudi Arabia; ^2^Department of Pediatrics, College of Medicine, Imam Mohammad Ibn Saud Islamic University (IMSIU), Riyadh, Saudi Arabia

## Abstract

**Objective:**

Parental recognition of children's developmental milestones has been correlated with more effective childcare strategies and overall better outcomes for children. However, the knowledge that parents have about children's development remains uncertain which reflects serious concerns about children's health in Saudi Arabia. Therefore, this study was undertaken to identify parents' knowledge levels about children's developmental milestones and the information sources they rely on for this knowledge. *Study Design*. We recruited 1471 parents aged ≥18 with at least one child under 14 and living in Riyadh to participate in an online survey between July of 2019 and January of 2020.

**Results:**

Most respondents showed a poor level of knowledge (80.0%) in all of four domains. However, mothers had more acceptable levels of knowledge (21.0%) than fathers (10.0%) (*p* ≤ 0.01). There was a significant association between knowledge and age at first child's birth; respondents who had their first child between 39 and 50 had the highest levels of knowledge (37.5%; *p* ≤ 0.01). In the four domains of development, parents were found to have the most knowledge about physical development (52.3%), followed by cognitive development (21.6%), social development (21.5%), and emotional development (21.2%). Only a few parents (5%) claimed to always rely on their healthcare professionals for information.

**Conclusion:**

There is a lack of appropriate knowledge about developmental milestones among parents, which reflects serious concerns about children's health in Saudi Arabia. Healthcare institutions and pediatricians currently play a minimal role in health education. Effective health education programs and strategies should be implemented to improve child development outcomes in the community.

## 1. Introduction

Children's early life experiences play an important role in shaping productive community members. This is especially true of the experiences that happen in the first five years of life, as this period is significant with regard to children's physical and mental development [1]. While children's development influences their interaction and social bonding with family and friends, literature has shown that parents' knowledge about children's development also has a major influence on children's interaction and development, and it is markedly associated with positive parenting efficacy [2–4]. A child's ability to roll over, crawl, stand, walk, and run are crucial milestones in neurological development, and parents' awareness of possible signs of delay is vital in ensuring proper child development [5, 6]. Due to parents being the main caregivers of children in their earlier years, parental awareness of developmental milestones helps them create a healthy environment, have appropriate expectations, and interact positively with their child [7–9]. It has previously been observed that parents with a low level of knowledge about child development demonstrated neglectful and abusive behaviors towards their children, and they were frustrated by the incompatibility of their expectations with their child's development [10]. Conversely, parents with a good level of knowledge showed high parental self-efficacy and competence [2, 8]. Studies done in Western countries have noted the importance of maternal knowledge about child development [11], as pediatric care and interventions often rely on a mother's observations for decision-making and medical counseling [11]. Furthermore, pediatricians often rely on parents for developmental milestone history; when parents are aware of such developmental steps, interaction with a pediatrician becomes more effective [4]. Parental knowledge about and competence in detection of developmental delays or abnormalities could lead to earlier interventions, which plays an important role in the promotion of a child's health and the prevention of diseases [8, 9, 12]. Public health education programs need to know a targeted population's baseline level of knowledge about children's developmental milestones to be able to apply an appropriate interventional approach that will result in maximum outreach [11, 12]. In Arabic literature, few studies have been conducted to measure parental knowledge of developmental milestones [7, 13, 14]. In Saudi Arabia, little has been reported about parental knowledge of developmental milestones, with only one study revealing pediatricians noticing a gap between maternal knowledge and children's health issues [15]. Considering the importance of this topic and the lack of information about it, the aim of this study is to identify knowledge levels about children's developmental milestones among parents in Saudi Arabia. We also aim to identify the sources of information that parents in Saudi Arabia rely on for gathering knowledge about children's developmental milestones. Hopefully, the results of this study can be the basis for planning public health programs to educate parents in Saudi Arabia about children's developmental milestones.

## 2. Materials and Methods

### 2.1. Study Design and Subjects

This cross-sectional study was conducted between July of 2019 and January of 2020 in Riyadh, Saudi Arabia. The desired population for this study were parents ≥ 18 years old with at least one child aged less than 14 years old living in Riyadh. With assistance from a public marketing organization and its access to the Saudi Telecom Company's database, 2000 adults were randomly selected and invited to participate in this study. An online survey was sent to participants through short message service (SMS) and included an explanation of the study and its purpose, with informed consent required prior to participation and completion of the questionnaire. This study's protocol was approved by the Institutional Review Board (IRB) of Imam Mohammad Ibn Saud Islamic University (IMSIU).

### 2.2. Sample Size

According to the Saudi General Authority for Statistics, the number of married Saudi adults who lived in Riyadh in 2017 was 1,925,179. Based on an acceptable margin of error equal to 4% and a design effect of 2 with 95% confidence, a sample size of at least 1200 was required: in our study, the total number of parents included in the analysis was 1471. The sample size was calculated using Epi Info version 7.2.3.1.

### 2.3. Data Collection Tool

With permission from Rikhy et al., the questionnaire for this study was adapted from one that had been designed to gauge adults' knowledge of children's developmental milestones in Alberta, Canada [8]. The questionnaire was translated into Arabic through a process that included cross-cultural validation and revision by a translation committee at IMSIU in Riyadh, Saudi Arabia, and a senior pediatrician. The self-administrated questionnaire included 17 items assessing parents' knowledge of children's developmental milestones in four domains: physical development (four questions), cognitive development (three questions), social development (five questions), and emotional development (five questions). A total of seven questions concerning parents' reliance on certain resources and the frequency of using each resource were also included. Demographic variables were obtained through 17 items and included age, gender, nationality, educational level, socioeconomic status, family censusing, and residence history. A pilot study was carried out with 10 participants to ensure the reliability and validity of the survey and address misapprehensions and discrepancies. Assessment of parents' knowledge about children's early life (from birth to 6 years old) developmental milestones was the primary objective. Selected milestones included observable events such as crawling, counting, and showing empathy. The participants' knowledge levels were categorized as “excellent” if they obtained a score of ≥75%, “good” if they obtained a score of between 50% and 75%, “fair” if they obtained a score of between 40% and 50%, and “poor” if they obtained a score of ≤39%. Participants who obtained a score of ≥50% for each developmental domain were considered to have an acceptable level of knowledge.

### 2.4. Data Analysis

Statistical Package for the Social Sciences (SPSS) version 24 was used for the analysis. Descriptive analysis was presented by frequency distributions and percentages. Cross tabulation and a chi-squared test of independent analysis were used at the bivariate level of analysis to examine any associations between respondents' demographic variables and their levels of knowledge. At the multivariate level of analysis, binary logistic regression was used to determine and compare the unadjusted and adjusted odds ratio of significant variables with regard to level of knowledge. A *p* value of ≤0.05 was considered statistically significant.

## 3. Results

A total of 1471 parents participated in the study (73.55% response rate). Mothers represented 65.4% (*n* = 962) and fathers 34.6% (*n* = 509) of the study sample. Almost all of the participating parents were married (97%; *n* = 1427). Most of the participants were Saudi (96.5%; *n* = 1420) and living in Riyadh (74%; *n* = 1088). More than half of the study sample were between the ages of 35 and 50 (60%; *n* = 882). The majority of the parents had their first child between the ages of 15 and 26 (64%; *n* = 941). Most of the participants were workers (64.4%; *n* = 947) with middle economic status (70%; *n* = 1035) and have had a stable residency for the past five years (52.1%; *n* = 767). With regard to education levels, 62.3% had completed a university degree, 24.6% had completed high school or less, and 12.8% had a masters or doctorate degree. Details of other characteristics of the participants are presented in Table 1.

Most of the respondents (80.0%) showed a “poor” level of knowledge; 14.2% had a “fair” level of knowledge, 2.3% showed a “good” level, and only 0.1% had an “excellent” level of knowledge in all of four developmental domains (Table 2).

Table 3 presents the cross tabulation and chi-squared test of independent results of the demographic variables and knowledge levels. The results showed a significant statistical relationship between gender and level of knowledge (*p* ≤ 0.01): Females had a more acceptable level of knowledge (21.0%) than males (10.0%). It was also found that respondents who had their first child between the ages of 39 and 50 had the highest levels of knowledge compared to other age groups (37.5%; *p* ≤ 0.01). In addition, the chi-squared test of independent results revealed a statistically significant association between job status and knowledge levels; 22.6% of those who were unemployed had an acceptable level of knowledge, whereas 19.3% of retirees, 18% of students, and 14.9% of employed parents had an acceptable level of knowledge (*p* ≤ 0.05). Moreover, there was a significant difference between age of first child and level of knowledge; 18.8% of respondents whose first child was seven or older had an acceptable level of knowledge, in comparison to 13.2% whose first child was between one day and six years old (*p* ≤ 0.05).

Table 4 shows an adjusted odds ratio of significant variables. It was found that females were two times more likely to have an acceptable level of knowledge (OR = 2.299; CI 1.572–3.361) compared to males. The results also showed that those who had their first child between the ages of 39 and 50 were five times more likely to have an acceptable level of knowledge (OR = 5.494; CI 1.242–24.306) than those who had their first child between the ages of 15 and 26. Furthermore, the results showed that respondents whose first child was between 7 and 16 (54%) were more likely to have an acceptable level of knowledge (OR = 1.543; CI 1.070–2.226) than respondents whose first child was between one day and six years old.

In the four domains of development, parents had the most knowledge of physical development, with 52.3% of the answers being correct; this was followed by cognitive development (21.6%), social development (21.5%), and emotional development (21.2%), as shown in Table 5.

The most “always” used source of information among parents was a family relative (17%), followed by internet websites (14.2%), books and parenting magazines (7.1%), medical physicians and pediatricians (5%), TV shows (3.6%), and parenting courses (3.5%). Further details of “often,” “rarely,” and “never” used sources can be seen in Table 6.

## 4. Discussion

Parents and other caregivers play an essential role in the observation and assessment of their children's development. The knowledge of different developmental domains and their associated milestones is crucial, as it enhances effective parenting and thereby improves child outcomes. In addition, it helps parents recognize developmental warning signs early and seek professional advice. To our knowledge, this is the first report that is aimed at assessing Saudi parents' awareness levels of developmental milestones among different pediatric age groups.

Our findings indicate that most Saudi parents were not knowledgeable about developmental milestones in children. Four-fifths of the participants received a “poor” score on the overall assessment, as they answered less than 40% of the total questions correctly. It is interesting to note that a similar level of knowledge was reported among Albertan adults [8], while higher scores were reported among other Arab populations [7, 13]. Such differences in results could be related to a variety of reasons. First, we used only multiple-choice questions in our questionnaire instead of including true and false statements. Second, the assessment and calculation of scores were based on all of the domains together rather than each one individually. Moreover, we assessed all of the pediatric age categories instead of targeting a specific age group. Finally, we included male parents in our assessment, and they scored lower compared to females.

A possible reason for the disappointing level of awareness from our report might be explained by the information sources of our participants. Family relatives and internet websites were the most common sources for knowledge and facts about normal child development, and only 5% of the participants claimed to “always” rely on their physicians as a source of information, while almost a third stated that they “sometimes” do. It can be inferred from these results that pediatric healthcare professionals need to provide parental guidance and address pediatric development specifically during well-child visits.

Despite most of the participants performing poorly in the overall assessment, their knowledge of motor development was acceptable, as more than half of these questions were answered correctly. Knowledge about other domains, including emotional, cognitive, and social, was particularly poor, which is similar to the findings mentioned by Rikhy et al. [8]. It is worrisome that a delay in the identification and diagnosis of some behavioral and developmental disorders could happen as a consequence. Parents must be able to obtain the services and support they need as early as possible; thus, this lack of specific knowledge is an essential reason for the need for education at the public level. The Ministry of Health should consider targeting these domains specifically when designing effective parenting programs and educational materials for Saudi parents.

Most of the existing studies about normal child development focus mainly on mothers [4, 7, 11, 13, 14]. In our study, we tried to identify the knowledge gaps of both parents, and we found that mothers are twice as knowledgeable as fathers, which could be related to more frequent contact with their children [16]; similar gender-related findings were reported in the literature among Western populations [4, 8]. Interestingly, despite what has been mentioned about the long-term negative consequences of delayed motherhood [17], we found that postponement of childbearing was associated with a better level of parental knowledge of developmental milestones. This finding could be helpful when assessing the benefits and risks of delaying parenthood. In our study, we were unable to identify any significant association between education level and parental awareness of child development; a similar lack of association was mentioned in the Albertan study [8]. However, previous regional reports among other Middle Eastern and Arabian Gulf countries have suggested that maternal education could be a predictor of good maternal knowledge of developmental milestones [7, 13, 14]. Reasons behind these inconsistent results include the differences of sample sizes, studied populations, study designs, and others.

We have encountered several limitations of the present study that should be considered in further exploration of the topic. First, the participation was limited to those who could be reached by phone and read Arabic, had an internet connection, and were willing to spend a few minutes filling out the electronic questionnaire. In addition, most of the study sample consisted of those of middle and high economic statuses, which might have limited the scaling of the knowledge levels among those with lower incomes. The study survey was restricted in the number of questions that was asked for each developmental domain, and there was a difference in the number of questions dedicated to each domain, which might lessen the precision of the findings. Accordingly, modifying and balancing the number of questions between all different domains would help in scaling the knowledge gap and refining understanding in future studies. Despite these limitations, the results of the current study have expanded upon previous research by including fathers who contribute to childcare during the phases of development.

The results of this study emphasize the need for further educational programs about developmental milestones targeting expecting and current parents. Moreover, it is important to provide reliable and available information related to the subject for both parents and the general public in Saudi Arabia.

## 5. Conclusion

The early years of a child's life are crucial to their development and their future social, physical, cognitive, and mental health. Moreover, as the responsibility of childcare in most cultures and communities during this critical period is that of the parents, their knowledge about developmental milestones influences the nature and quality of care the child receives. However, the majority of parents had undesirable levels of knowledge and were incapable of correctly answering questions related to children's developmental milestones; gaps in knowledge about child development exist among parents, although females were more competent in identifying milestones than their male counterparts. Despite limited overall understanding of developmental milestones, parents' awareness of motor development exceeded that of emotional, cognitive, and social developments. Furthermore, this study showed that parents do not usually seek advice about child development from reliable sources. Therefore, healthcare providers must be adequately informed about child development and take the initiative to disseminate accurate information to primary caregivers. Moreover, the need for strategies to improve the awareness of developmental milestones among parents to support optimal child development and achieve maximum outcomes must be highlighted.

## Figures and Tables

**Table 1:** Sociodemographic characteristics of the study population.

	*n* ^1^ (%)
Gender	
Female	962 (65.4)
Male	509 (34.6)
Nationality	
Non-Saudi	51 (3.5)
Saudi	1420 (96.5)
Age	
19-34	482 (32.8)
35-50	882 (60.0)
51-66	107 (7.3)
Age at first childbirth	
15-26	941 (64.0)
27-38	522 (35.5)
39-50	8 (0.5)
Residency	
Urban	1088 (74.0)
Rural	383 (26.0)
Number of people living in home	
≤5	724 (49.2)
≥6	747 (50.8)
Job status	
Retired	109 (7.4)
Student	61 (4.1)
Unemployed	354 (24.1)
Working	947 (64.4)
Economic status	
Low	54 (3.7)
Middle	1035 (70.4)
High	382 (26.0)
Moved in the past year	
No	1206 (82.0)
Yes	265 (18.0)
Number of times moved in the past five years	
None	767 (52.1)
One time	493 (33.5)
Two times	141 (9.6)
Three or more times	70 (4.8)
Level of education	
Diploma, high school or less	366 (24.9)
College/university	917 (62.3)
Higher education (doctorate or master)	188 (12.8)
Marital status	
Married	1427 (97.0)
Divorced	32 (2.2)
Widowed	12 (0.8)
Number of children under 14 years	
1	448 (30.5)
2	512 (34.8)
3	305 (20.7)
4	148 (10.1)
≥5	58 (3.9)
Age of first child	
1 day to less than 7 years	418 (28.4)
7 years to less than 17 years	538 (36.6)
17 years and more	515 (35.0)
Gender of first child	
Female	701 (47.7)
Male	770 (52.3)
Residence of children with their parents	
Never	7 (0.5)
Partial	39 (2.7)
Always	1425 (96.9)
Children with special needs	
No	1393 (94.7)
Yes	78 (5.3)

^1^Number of the participants.

**Table 2:** Parents' level of knowledge about developmental milestones.

Level of knowledge	*n* ^1^ (%)
Poor	1218 (80.0)
Fair	216 (14.2)
Good	35 (2.3)
Excellent	2 (0.1)

^1^Number of the participants.

**Table 3:** Association between sociodemographic characteristics and knowledge of respondents about developmental milestones.

Demographic	Level of knowledge		*χ* ^2^	*p* value
	Not accepted *n*^1^ (%)	Accepted *n*^1^ (%)		
Gender				
Female	760 (79.0)	202 (21.0)	28.171	*p* ≤ 0.001^∗^
Male	458 (90.0)	51 (10.0)		
Nationality				
Non-Saudi	43 (84.3)	8 (15.7)	0.085	*p* ≥ 0.05
Saudi	1175 (82.7)	245 (17.3)		
Age				
19-34	405 (84.0)	77 (16.0)	1.435	*p* ≥ 0.05
35-50	722 (81.9)	160 (18.1)		
51-66	91 (85.0)	16 (15.0)		
Age at first childbirth				
15-26	761 (80.9)	180 (19.1)	10.038	*p* ≤ 0.05^∗^
27-38	452 (86.6)	70 (13.4)		
39-50	5 (62.5)	3 (37.5)		
Residency				
Urban	890 (81.8)	198 (18.2)	2.930	*p* ≥ 0.05
Rural	328 (85.6)	55 (14.4)		
Number of people living in home				
≤5	613 (84.7)	111 (15.3)	3.492	*p* ≥ 0.05
≥6	605 (81.0)	142 (19.0)		
Job status				
Student	50 (82.0)	11 (18.0)	11.153	*p* ≤ 0.05^∗^
Working	806 (85.1)	141 (14.9)		
Retired	88 (80.7)	21 (19.3)		
Unemployed	274 (77.4)	80 (22.6)		
Economic status				
Low	47 (87.0)	7 (13.0)	0.983	*p* ≥ 0.05
Middle	852 (82.3)	183 (17.7)		
High	319 (83.5)	63 (16.5)		
Moved in the past year				
No	997 (82.7)	209 (17.3)	0.080	*p* ≥ 0.05
Yes	221 (83.4)	44 (16.6)		
Number of times moved in the past five years				
None	631 (82.3)	136 (17.7)	0.788	*p* ≥ 0.05
One time	408 (82.8)	85 (17.2)		
Two times	120 (85.1)	21 (14.9)		
Three or more times	59 (84.3)	11 (15.7)		
Level of education				
Diploma, high school or less	297 (81.1)	69 (18.9)	3.211	*p* ≥ 0.05
College/university	759 (82.8)	158 (17.2)		
Higher education (doctorate or master)	162 (86.2)	26 (13.8)		
Marital status				
Married	1186 (83.1)	241 (16.9)	3.292	*p* ≥ 0.05
Divorced	23 (71.9)	9 (28.1)		
Widowed	9 (75.0)	3 (25.0)		
Number of children under 14 years				
1	381 (85.0)	67 (15.0)	6.808	*p* ≥ 0.05
2	428 (83.6)	84 (16.4)		
3	238 (78.0)	67 (22.0)		
4	122 (82.4)	26 (17.6)		
≥5	49 (84.5)	9 (15.5)		
Age of first child				
1 day to 6 years	363 (86.8)	55 (13.2)	6.697	*p* ≤ 0.05^∗^
7 years to 16 years	437 (81.2)	101 (18.8)		
17 years and more	418 (81.2)	97 (18.8)		
Gender of first child				
Female	583 (83.2)	118 (16.8)	0.126	*p* ≥ 0.05
Male	635 (82.5)	135 (17.5)		
Residence of children with their parents				
Never	7 (100)	0	1.474	*p* ≥ 0.05
Partial	32 (82.1)	7 (17.9)		
Always	1179 (82.7)	246 (17.3)		
Children with special needs				
No	1156 (83.0)	237 (17.0)	0.635	*p* ≥ 0.05
Yes	62 (79.5)	16 (20.5)		

^1^Number of the participants; ^∗^statistically significant.

**Table 4:** Adjusted odds ratios (OR) and 95% confidence intervals (95% CI) of predictors for participants' knowledge of developmental milestones.

Demographic	OR	95% CI	*p* value
Gender			
Male	Reference		
Female	2.299	1.572–3.361	*p* ≤ 0.001^∗^
Age at first childbirth			
15-26	Reference		
27-38	0.984	0.698–1.388	*p* ≥ 0.05
39-50	5.494	1.242–24.306	*p* ≤ 0.05^∗^
Age of first child			
1 day to 6 years	Reference		
7 years to 16 years	1.543	1.070–2.226	*p* ≤ 0.05^∗^
17 years and more	1.474	0.992–2.190	*p* ≥ 0.05

OR: odd ratio; CI: confidence interval; ^∗^statistically significant.

**Table 5:** Proportion of correct and incorrect responses to questions in each developmental domain.

Developmental domain or milestone	Correct answer	Correct responses *n*^1^ (%)	Incorrect responses *n*^1^ (%)
*Physical development*		*52.3% of the questions*	
Walk	12-18 months	994 (67.6)	477 (32.4)
Crawl	6-12 months	1004 (68.3)	467 (31.7)
Reach for objects	4-6 months	755 (51.3)	716 (48.7)
Dress themselves	24-36 months	324 (22.0)	1147 (78.0)
*Cognitive development*		*21.6% of the questions*	
Engage in pretend/fantasy play	12-18 months	182 (12.4)	1289 (87.6)
Follow simple instructions	12-18 months	306 (20.8)	1165 (79.2)
Begin counting	24-36 months	468 (31.8)	1003 (68.2)
*Social development*		*21.5% of the questions*	
Parallel play	18-24 months	287 (19.5)	1184 (80.5)
Share toys	36-60 months	356 (24.2)	1115 (75.8)
Play alone for 1 hour	36-60 months	282 (19.2)	1189 (80.8)
Have best friends	60-72 months	342 (23.2)	1129 (76.8)
Show empathy	60-72 months	316 (21.5)	1155 (78.5)
*Emotional development*		*21.2% of the questions*	
Exert independence	12-18 months	31 (2.1)	1440 (97.9)
Recognize others' emotions	6-12 months	405 (27.5)	1066 (72.5)
Differential cries	4-6 months	339 (23.0)	1132 (77.0)
Bond with caregiver	4-6 months	382 (26.0)	1089 (74.0)
Advocate for fairness	60-72 months	403 (27.4)	1068 (72.6)

^1^Number of the participants.

**Table 6:** Sources of information used by parents regarding developmental milestones.

	*n* ^1^	Percentage (%)
Medical physician & pediatrician		
Never	623	42.4
Rarely	250	17.0
Yes, sometimes	524	35.6
Yes, always	74	5.0
Family relatives		
Never	333	22.6
Rarely	205	13.9
Yes, sometimes	679	46.2
Yes, always	254	17.3
Books and parenting magazines		
Never	566	38.5
Rarely	277	18.8
Yes, sometimes	524	35.6
Yes, always	104	7.1
Internet websites		
Never	401	27.3
Rarely	272	18.5
Yes, sometimes	579	39.4
Yes, always	219	14.9
*Social media broadcasts*		
Never	651	44.3
Rarely	350	23.8
Yes, sometimes	418	28.4
Yes, always	52	3.5
*Parenting courses*		
Never	1018	69.2
Rarely	192	13.1
Yes, sometimes	209	14.2
Yes, always	52	3.5
*Television shows*		
Never	562	38.2
Rarely	401	27.3
Yes, sometimes	455	30.9
Yes, always	53	3.6

^1^Number of the participants.

## Data Availability

The data used to support the findings of this study are included within the article.
